# Epidermoid cyst in an intrapancreatic accessory spleen in the pancreas head: a case report

**DOI:** 10.1186/s12876-020-01540-4

**Published:** 2020-11-20

**Authors:** Hyo Jung Ko, Jae Ryong Shim, Tae Beom Lee, Byung Hyun Choi, Jung-Hee Lee, Je Ho Ryu, Kwangho Yang

**Affiliations:** 1 Division of Hepato-Biliary-Pancreatic Surgery and Transplantation, Department of Surgery, Pusan National University Yangsan Hospital, Yangsan, Gyeongsangnam-do 50612 Republic of Korea; 2 Department of Pathology, Pusan National University Yangsan Hospital, Yangsan, Republic of Korea; 3 Research Institute for Convergence of Biomedical Science and Technology, Pusan National University Yangsan Hospital, Yangsan, Gyeongsangnam-do 50612 Republic of Korea

**Keywords:** Epidermoid cyst in an intrapancreatic accessory spleen, Pancreatic cystic tumor, Case report

## Abstract

**Background:**

An epidermoid cyst in an intrapancreatic accessory spleen (ECIPAS) in the pancreas head is an extremely rare condition. The natural course of this condition is not well known, and it is difficult to diagnose before surgery due to the lack of specific imaging findings.

**Case presentation:**

A tumor was found in the head of the pancreas in a 68-year-old man with abdominal distension and discomfort. Magnetic resonance imaging (MRI) suggested a malignant tumor, such as a colloid cancer. The tumor was removed surgically, with pathologic examination showing that it was an ECIPAS.

**Conclusion:**

ECIPAS cannot be easily distinguished from other pancreatic cystic tumors, making it necessary to include ECIPAS in the differential diagnosis of these tumors. Unnecessary surgical resection may be avoided by more accurate preoperative diagnosis based on clinical and imaging characteristics.

## Background

Accessory spleen is found in about 10% of the general population, with 20% of accessory spleens located in or attached to the tail of the pancreas [1]. Epidermoid cysts account for about 10% of benign and non-parasitic cysts of the spleen [2]. However, epidermoid cyst in an intrapancreatic accessory spleen (ECIPAS) is very rare, with a prevalence of 1.7% in the general population [1]. Differential diagnosis of cystic tumors of the pancreas is difficult because they often show findings similar to other conditions on imaging modalities [3]. It is also difficult to differentiate ECIPAS from other cystic tumors, such as pancreatic pseudocysts, serous cystic neoplasms, mucinous cystic neoplasms, intraductal papillary mucinous neoplasms, and lymphoepithelial cysts, or from solid pancreatic tumors [4]. Of 56 patients with ECIPAS described to date, only five have been diagnosed accurately before surgery.

Herein we report a 68-year-old man with an ECIPAS on the head of the pancreas and review the literature on this rare condition.

## Case presentation

A 68-year-old man visited a local physician because of abdominal distension and abdominal discomfort of several months’ duration. Abdominal ultrasound and computed tomography showed a mass lesion on the head of the pancreas. He was admitted to our hospital for further examination and treatment. The patient had no specific medical or surgical history. He was a 40 pack-year smoker, but had stopped drinking alcohol about 30 years earlier. Family history included a sister with breast cancer. Physical examination at admission showed mild abdominal distension, no tenderness or rebound tenderness, and no palpable abdominal mass. Peripheral blood tests showed a white blood cell count of 11,520/mm^3^, a hemoglobin concentration of 15.5 g/dl, and a platelet count of 252,000/mm^3^. Most blood chemistry tests were within the normal range, including concentrations of AST (49 IU/L), ALT (94 IU/L), ALP (191 IU/L), total bilirubin (0.5 mg/dl), total protein (7.5 g/dl), albumin (4.4 g/dl), amylase (55 IU/L), and lipase (18 U/L). Tumor marker concentrations were normal, with CEA of 1.29 ng/ml and CA 19–9 of 14.8 U/ml.

**Fig. 1:**
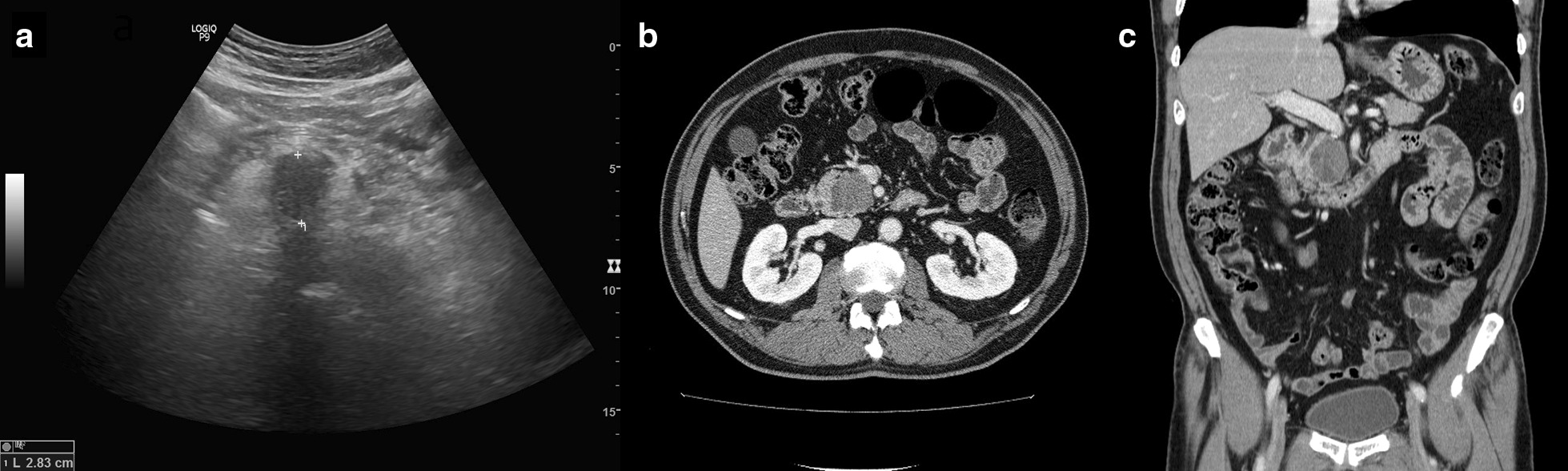
**a** Ultrasonography demonstrates a 2.8 cm sized hypoechoic mass lesion in the pancreatic head. **b**, **c** Computed tomography scan showing a 3.6 × 3 × 3.3 cm sized hypodense mass lesion in the pancreatic head region

**Fig. 2:**
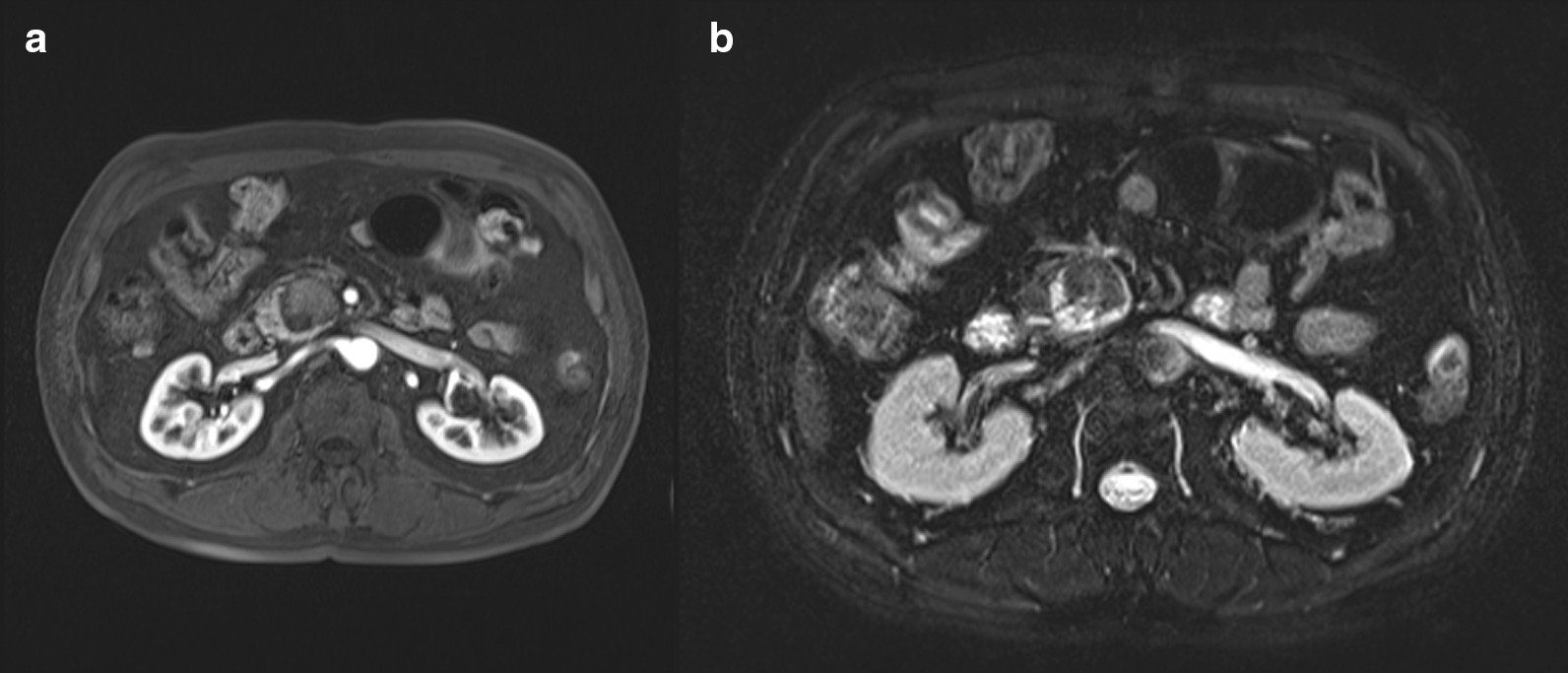
Magnetic resonance imaging showing a cystic lesion with low signal intensity (SI) and high SI focus on T1WI (**a**) and high SI with low SI focus on T2WI (**b**)

Abdominal ultrasound at the local clinic showed a hypoechoic mass in the pancreas head, and abdominal computed tomography (CT) revealed a hypoechoic mass in the pancreas head measuring 3.6 × 3 × 3.3 cm, suggesting a high probability of pancreatic cancer (Fig. 1). He was admitted for further examination and surgical treatment, if necessary. Magnetic resonance imaging (MRI) of his pancreas showed a 3 cm sized, well-defined ovoid mass in the pancreas head, with low signal intensity (SI) and high SI focus on T1-weighted images (T1WI) and high SI with low SI focus on T2-weighted images (T2WI) (Fig. 2). Most portions of the mass showed lack of enhancement, although subtle enhancing rinds and threads, as well as restricted diffusion, were present at the periphery of the mass, and the pancreatic ducts were not dilated. The patient was suspected of having a colloid carcinoma, or an acinar cell carcinoma, and the possibility of a solid pseudopapillary neoplasm was also considered. Preoperative bone scan and non-enhanced CT of the chest showed no evidence of distant metastases.

**Fig. 3:**
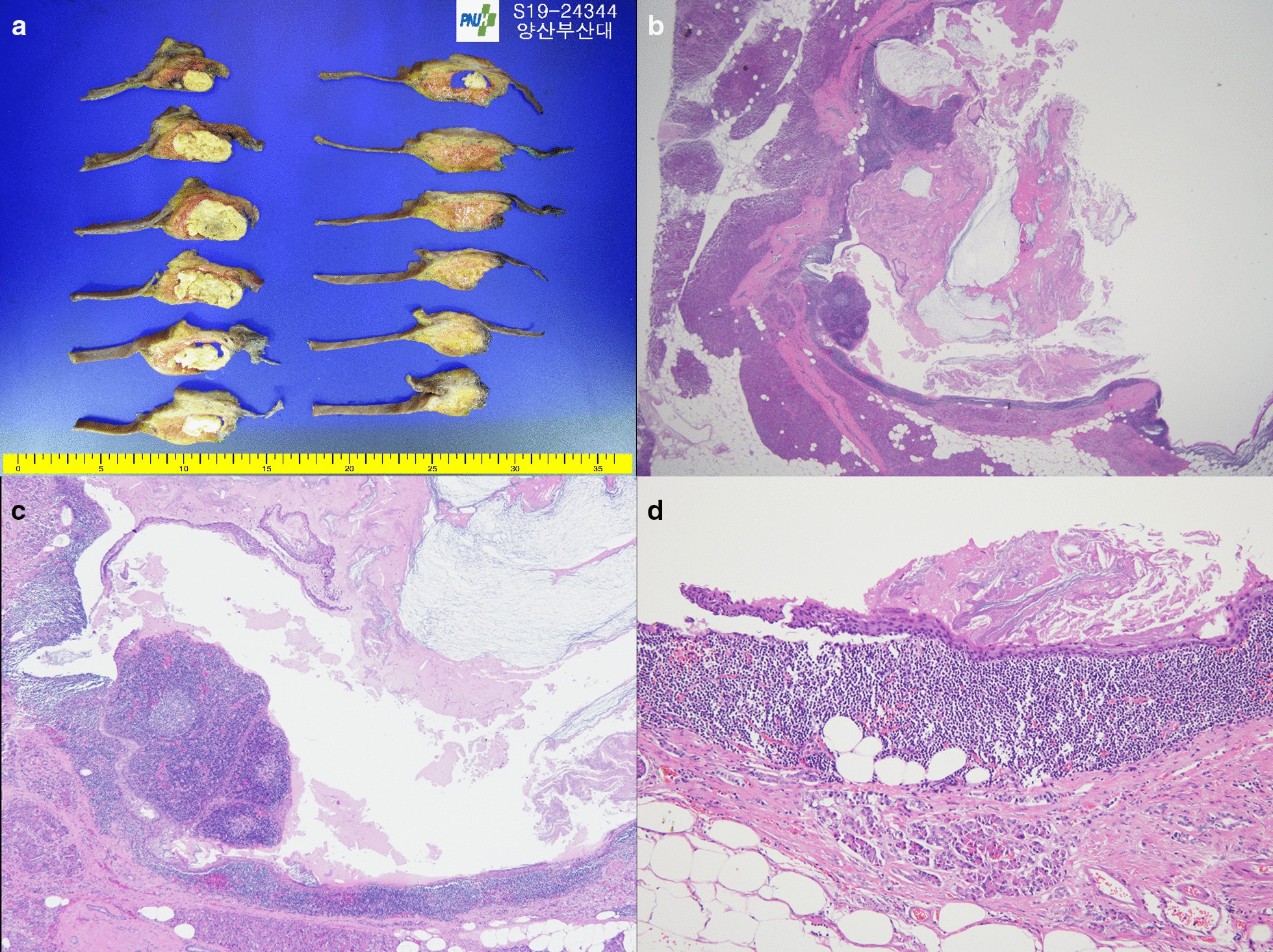
Pathologic features of the tumor. **a** The resected specimen consisted of a well-defined brown-white ovoid unilocular cystic mass in the pancreas, measuring 3.7 × 3.5 × 2.6 cm and filled with yellow-white friable material. **b**–**d** Microscopic examination showing that the intrapancreatic accessory spleen contains typical white and red pulp. **H**–**e**; **b**, × 12.5; **c** × 40) and that the cystic lesion was lined by multi-layered epithelium and contained keratin (**d**; **h**–**e** × 100)

The patient underwent a pylorus preserving pancreaticoduodenectomy (PPPD) to remove the tumor. Intraoperative examination of the resected tissue showed a well-defined brown-white ovoid unilocular cystic mass in the head of the pancreas, measuring 3.7 × 3.5 × 2.6 cm and filled with yellow-white friable material. Microscopic findings showed cysts consisting of multiple layers of squamous epithelium, which were not large in size due to the mass effect of the cyst. However, splenic tissue with specific white and red medulla was observed, leading to a final pathological diagnosis of ECIPAS (Fig. 3).

## Discussion and conclusions

Accessory spleen is relatively common, observed in 10% of the general population [1]. The most frequent location is the splenic hilum (80%), followed by the pancreas (17%) [2]. Specific histopathological examinations of epidermoid cysts showed that they are unilocular or multilocular cystic masses, with inner walls consisting of keratinized or non-keratinized stratified squamous epithelium inside normal splenic tissue. Epidermoid cysts differ from dermoid cysts in that they do not have hair or skin appendages, and they differ from lymphoepithelial cysts in that there is no lymphocyte infiltration [5].

ECIPAS is a very rare disease. Since the first patient was described in 1980 [6], 47 articles to date in the English language literature have described 56 patients with ECIPAS. Most of these cysts have been found in young people, whereas the present patient was a 68-year-old man. More than 50% of these cysts have been discovered incidentally, although the incidence of ECIPAS has increased with the development of imaging technologies such as CT, MRI, and endoscopic ultrasound. These modalities usually show a unilocular or multilocular cystic mass in the pancreas, with a thick wall or a solid substance in the cyst having similar SI. In most patients, serum concentrations of CA19-9 are elevated due to the presence of squamous epithelial cell tissue in these epidermoid cysts [4, 7]. Thus, prior to surgery, it is difficult to differentiate between an ECIPAS and a cystic pancreatic malignancy.

An accessory spleen is mostly caused by incomplete fusion of the mesenchymal buds during embryogenesis. It may be dragged by splenic ligaments to ectopic locations, and is most often located in the vicinity of the splenic hilum. However, it may also occur in the pancreatic tail, greater omentum, mesentery of the small intestine, and pouch of Douglas. Trauma or auto-transplantation after splenectomy can also lead to the development of accessory spleen. However, the patient in this study had no history of trauma or abdominal surgery. Therefore, failure of splenic anlage fusion may have been the cause of the accessory spleen in this patient [8, 9].

All previous ECIPAS reported to date have been found in the pancreas tail, suggesting that the epithelium of ECIPAS is derived from the pancreatic duct [10]. By contrast, the ECIPAS in this patient was found in the pancreatic head. The only previous case reported in English was by Landry et al. [11]. The exact embryological mechanism for the formation of an accessory spleen in the pancreas head will need to investigated in a future study.

Eight patients with ECIPAS, six men and two women, ranging in age from 32 to 68 years, have been reported in Korea to date. Seven underwent surgical resection for suspicion of other diseases, such as pancreatic cancer or pancreatic cystic neoplasm. One Korean patient, however, was diagnosed with ECIPAS preoperatively. Moreover, only one of all patients correctly diagnosed preoperatively was followed up without resection in the English language literature.

ECIPAS is difficult to diagnose preoperatively because it has no specific radiologic findings and is therefore likely to be misdiagnosed as a pancreatic cystic neoplasm [12]. The accessory spleen surrounding the cyst may be key to accurate preoperative diagnosis, with the difference in contrast enhancement between the spleen parenchyma and the parenchymal component of the lesion being important in the differential diagnosis of pancreatic cystic masses [13]. CT and/or MRI showed solid components in 8 of 13 patients with ECIPAS, with imaging of the solid component similar to that of the spleen [14]. These findings suggested that the presence of a relatively large amount of accessory spleen tissue in the pancreas may enable a correct preoperative diagnosis by careful imaging examination. Because the solid component of mass and the spleen have a similar degree of contrast enhancement on CT and similar intensity on MRI, radiographic imaging can be a tool for diagnosis of ECIPAS prior to surgery. Unfortunately, similar to previous findings[12], the amount of accessory spleen was lower in the present patient, preventing an accurate diagnosis by preoperative imaging. Therefore, ECIPAS should be considered in the differential diagnosis of pancreatic cystic masses, although most cannot be correctly diagnosed until pathologic examination after surgical resection.

Endoscopic ultrasound-guided fine-needle aspiration (EUS-FNA) has played an important role in the triage of pancreatic lesions and has led to the accurate diagnosis of benign disease and avoidance of unnecessary operations. EUS-FNA may also be helpful for the diagnosis of ECIPAS [15, 16]. However, its diagnostic ability has not yet been determined because of the rare incidence of this condition [17]. When it is suspected in imaging tests, ECIPAS can be reliably diagnosed by performing a cytologic smear or a biopsy if an adequate amount of solid splenic tissue can be sampled by EUS-FNA [4, 18]. On the other hand, if the splenic tissue is small and only the surrounding epidermoid cystic fluid can be aspirated, there is no advantage to performing EUS-FNA [19]. In this case, colloid (mucinous non-cystic) carcinoma of the pancreas was most strongly suspected on preoperative CT and MRI. In this study, radiologists suggested an acinar cell carcinoma or a solid pseudopapillary tumor as a differential diagnosis, all which are indication of surgical intervention. CT and MRI are useful modalities for the diagnosis of colloid carcinoma of the pancreas. Although EUS-FNA may help to confirm a diagnosis of colloid carcinoma of the pancreas, aspiration or biopsy may lead to spreading of the primary tumor. Therefore, we decided not to perform preoperative EUS-FNA. In addition, as is widely known, it can cause hemorrhage, and infections such as pancreatitis [20, 21]. Thus, EUS-FNA or biopsy should be done carefully considering the advantages and disadvantages of the procedure depending on each patient.

To date, ECIPAS has been considered benign and to be located in the pancreas tail. Surgery has therefore consisted of open or laparoscopic distal pancreatectomy, with or without splenic preservation. No deaths have been reported intraoperatively or shortly after surgery.

ECIPAS is an extremely rare disease entity, with no standard criteria for preoperative diagnosis. Pathologic confirmation requires surgical removal. This report describes a 68-year-old man with a pancreatic mass in the head of the pancreas. Preoperative CT and MRI suggested a pancreatic malignancy, but pathologic examination after PPPD resulted in a diagnosis of ECIPAS. Current radiological and laboratory methods are limited in their ability to diagnose ECIPAS, indicating the need to assess the diagnostic ability of other imaging methods, such as superparamagnetic iron oxide-based MRI or ^99m^TC-Sn-colloid scintigraphy. Accurate diagnosis may help determine appropriate treatment and could reduce unnecessary surgical resection of the pancreas.

## Data Availability

This case report contains clinical data from the electronic medical record in the Pusan National University Yangsan Hospital. The datasets used during the current study are available from the corresponding author on reasonable request.

## Abbreviations

ECIPAS: Epidermoid cyst in an intrapancreatic accessory spleen
MRI: Magnetic resonance imaging
CT: Computed tomography
SI: Signal intensity
T1WI: T1-weighted images
T2WI: T2-weighted images
PPPD: Pylorus preserving pancreaticoduodenectomy
EUS-FNA: Endoscopic ultrasound-guided fine-needle aspiration
